# Longitudinal patterns of provided oral healthcare services to Dutch young patients: An observational study

**DOI:** 10.1371/journal.pone.0299470

**Published:** 2024-02-23

**Authors:** Riët Hummel, Joost den Boer, Geert van der Heijden, Wil van der Sanden, Josef Bruers

**Affiliations:** 1 Department of Oral Public Health (OPH), Academic Centre for Dentistry Amsterdam (ACTA), University of Amsterdam and Vrije Universiteit Amsterdam, Amsterdam, The Netherlands; 2 Zilveren Kruis, Zeist, The Netherlands; 3 KNMT, Royal Dutch Dental Association, Utrecht, The Netherlands; 4 Department of Dentistry—Quality and Safety of Oral Healthcare, Radboud University Medical Center, Radboud Institute for Health Sciences, Nijmegen, The Netherlands; Shahid Beheshti University of Medical Sciences School of Dentistry, ISLAMIC REPUBLIC OF IRAN

## Abstract

General dental practitioners (GDPs) differ in the preventive and curative care they provide to their young patients. This may be related to variation in the caries risk of patients, but also to differing opinions among GDPs about ’proper care’. Longitudinal data offers the possibility to make care patterns of GDPs comparable and to reveal possible treatment variation between GDPs. GDPs who participated in this study delivered data on the oral healthcare services (OHS) they provided to young patients during the period 2013–2017. Subsequently, data from patients who received regular OHS for 4 to 5 years were used in the analyses. Based on this, longitudinal preventive and curative care patterns were distinguished. Patients were divided into 3 preventive care patterns: no prevention, occasional prevention, and regular prevention. Furthermore, 3 curative care patterns were distinguished: no curation, curation in 1 year, and curation in several years. These care patterns were then combined. In addition, patients were classified into caries risk categories based on the caries-related treatments they received over a 2-year period: low (no procedures), elevated (1 procedure), and high (2 or more procedures). The caries risk based on the first 2 years and the last 2 years in the dataset were combined into a longitudinal caries risk profile. The most frequent combined care pattern (35.8%) was no curation and occasional or regular prevention. The most common longitudinal caries risk profile was low at beginning and end (45.2%). Dental practices varied considerably in the distribution of curative and preventive care patterns. Thereby, no relationship was shown between curative care patterns and provided preventive care. There was also a large spread in the provided OHS within the various caries risk profiles. These diversities indicated treatment variation between GDPs, which is unwarranted if less or more care is provided than necessary.

## Introduction

The aim of providing oral healthcare services (OHS) is to individually achieve and maintain oral health [1]. In young patients, this mainly focuses on preventing the development of caries lesions or progression of existing lesions. In other words, keeping the caries risk low or lowering the caries risk.

However, there is uncertainty about the management of dental caries in young patients [2] and GDPs therefore differ in their treatment approach. These differences may of course be caused by a difference in the caries risk of patients. In addition, depending on their personal opinions about ’proper care’ for young patients, GDPs vary in the way they prevent and treat caries lesions [3, 4]. Treatment variation is warranted if it is caused by differences in caries risk and caries activity in patients but is unwarranted if it has a negative impact on the quality or costs of care [5, 6]. For instance, variation in preventive and curative treatment approaches can lead to overtreatment with iatrogenic damage and unnecessary costs as a result. On the other hand, it can lead to undertreatment, with unnecessarily higher caries incidence and burden of disease, and ultimately also higher costs as a result [7].

In the Netherlands, costs for OHS for patients under 18-years old are almost fully covered by a legally required standard health insurance and the payment system is fee-per-item. Therefore, the treatment approach of a GDP can be seen in the pattern of the claims data on the provided care. This care is often expressed as the proportion of patients with a certain procedure and/or as the mean number of a specific procedure per patient over a certain period [8–10]. This indicates the average care provided to a patient population in a certain period. Longitudinal data are needed to gain insight into the effects of that care. They also allow the description of caries-related care patterns over a longer period based on the number of years that preventive and curative care has been provided to a patient.

Another approach is to consider caries-related treatments as an approximation of caries risk. Mettes et al. [11] have proposed using the number of new caries lesions as a measure. Hummel et al. [12] have applied this idea by considering a claimed new restoration as an approximation of a new caries lesion. Based on the number of new restorations claimed in the baseline period of 2 years, they were able to divide their study population into 3 risk categories: low (no new restorations), moderate (1 new restoration) and high (2 or more new restorations). Three years later, the risk category was reassessed. In 21% of the participants this was higher and therefore, there was a deterioration of oral health, in 19% there was an improvement in oral health and in 60% the risk category was unchanged. Another way to represent this is to combine the caries risk at the beginning and the end of a period to form a longitudinal caries risk profile.

Treatment variation is usually shown with cross-sectional data, reflecting one moment in time. With longitudinal data, provided care could be compared over time. This would provide a more robust picture. A combination of provided preventive and curative care over time would allow for the examination of the relationship between them. As longitudinal care patterns and caries risk profiles were not described before, the aim of this observational study was to distinguish longitudinal caries-related patterns in Dutch children and adolescents and to reveal any treatment variation between GDPs over time. The research questions were:

- Can care patterns and caries risk profiles be distinguished among young patients based on dental procedures over a period of at least 4 to a maximum of 5 years?
- Are there differences in demographic characteristics of patients between the various care patterns and, if so, which?
- Are there differences between GDPs in provided preventive care to patients with similar curative care patterns and if so, which?
- Are there differences in provided care between the caries risk profiles and if so, which?

## Methods

For this study, GDPs delivered data on the care provided to young patients in their dental practice over a period of 5 years. The study was set up by the Department of Oral Public Health from the Academic Centre for Dentistry Amsterdam (ACTA) and was carried out in collaboration with the Royal Dutch Dental Association (KNMT), the Radboud University Medical Center and software supplier Vertimart (supplier dental software program Exquise).

### Participants and included patients

Dentists could participate in the study voluntarily. At the time of the data collection, they were working as GDPs, had been working in the same dental practice since 2013, had been using software program Exquise since 2013 and had been seeing at least 50 young patients (up to 17 years) for routine oral health examinations (ROEs). Participation was not possible for GDPs who were working in a dental practice that was part of a group of practices where the care provided and claimed could not be attributed to a specific practice location.

The participating GDPs delivered data on all care provided in their practice to patients born between 1 January 2000 and 31 December 2012. Hence, during the study period (January 2013-December 2017), patients were 0 to 17 years old and insured for the costs of OHS throughout this period. Subsequently, only regular patients were selected for inclusion in the analyses. Those were patients who, during the study period, had visited the participating dental practice in all 5 calendar years for at least 1 ROE with a gap of 1 year at the most, or in 4 consecutive calendar years (2013–2016 or 2014–2017) also with a gap of 1 year at the most (see the table in the results section).

### Data collection

Claims data was collected on the OHS provided over the period 1 January 2013 to 31 December 2017. This was done on behalf of the KNMT by the trusted data management and data protecting agency KBA in Nijmegen (https://www.kbanijmegen.nl/) via an application in the dental software program Exquise, which is an electronic patient record. The application was built in by its developer Vertimart. The data collection for research purposes was from 31 August 2020 to 19 February 2021. The medical Ethics Review Committee of VU University Medical Center waived the requirement for informed consent as they judged that the Medical Research Involving Human Subjects Act did not apply to this study (number 2019.551).

On behalf of the researchers, Vertimart invited the users of Exquise to participate in the study. If GDPs consented to participate, they sent data from their electronic patient records to the trusted data management and data protecting agency under non-traceable identification codes. This agency collected, administered, and saved the research data, ensuring that information could not be traced back to GDPs and patients in any way. The researchers were only given access to a database containing completely anonymous information.

### Data processing

The following information per patient was available: a (non-traceable) patient number, year of birth, gender, the income category linked to the 4-digit postal code of the home address and the procedure codes of all received OHS. Each procedure code contained additional information: a (non-traceable) code of the dental practice that provided the procedure, the treatment date and, if applicable, the tooth number.

To be able to interpret the data on the provided care, it was necessary to process them.

- The focus was mainly on radiographs made for caries diagnosis (bitewing radiographs). Periapical radiographs and bitewing radiographs had an identical procedure code. In some cases, a tooth number was added. Intraoral radiographs with tooth numbers were assumed to have been indicated for tooth-related reasons such as trauma or endodontic treatment. Therefore, these radiographs were excluded.
- Incidentally, a GDP claimed a total of 6 or 12 anterior one-surface-restorations on the same day for a patient. This mostly concerned patients who had also undergone orthodontic procedures or patients of GDPs who performed orthodontic treatments themselves instead of referring to an orthodontist. For this reason, it was assumed that these one-surface-restorations were (incorrectly) used for the attachment of a fixed orthodontic retainer and were therefore excluded.
- If the only tooth extraction(s) in the permanent dentition of a patient concerned 1 or more premolars, these were considered to have been indicated for orthodontic reasons and regarded as orthodontic procedures. Moreover, extractions of permanent teeth did not occur very often (11.8% of all tooth extractions), almost three-quarters of them were premolars. Caries in premolars is relatively uncommon in young patients [13]. This supported our assumption that these tooth extractions were part of orthodontic interventions.
- For sealants and Non-Operative Caries Treatment and Prevention in the primary dentition (NOCTP), it was considered more relevant whether those procedures were provided than the exact number of these procedures. The difference between patients who did or did not receive these procedures was larger than the differences between the patients who did receive these procedures. Their number per session was therefore recoded into none versus 1 or more.
- The number of years that a patient was included was determined by the number of years in which one or more ROEs had been executed. This was either 5 years if a patient had had at least 1 ROE in at least 4 study years and any missing year was an intermediate year, or 4 years if the missing year was 2013 or 2017 and the patient had at least 1 ROE in at least 3 of the other study years. If a patient had not had a ROE in the first or last study year (2013 or 2017), but did get other OHS in that specific year, those OHS were converted into missing. It was assumed that this was not yet or no longer a regular patient in that year (see the table in the results section).
- Per type of procedure, the numbers per year and during the entire study period were added per patient. This included 12 different procedures: ROEs, incidental consultations, intra-oral radiographs, sessions involving oral hygiene instruction, sessions involving professional tooth cleaning, sessions involving fluoride treatment, sessions involving 1 or more sealants, sessions involving NOCTP, restorations (including prefabricated crowns for primary teeth), tooth extractions, endodontic treatments, and orthodontic procedures (an overview is shown in Table 1).
- The year of birth was converted into age on 1 January 2013. That is age at the start of the study period.
- The determination of the family’s income category was based on the 4-digit postal code of the patient’s home address when the first claim was made. For this purpose, a table with income data per neighborhood in 2013 from Statistics Netherlands was used [14]. This table showed the number of inhabitants per neighborhood, the average income per inhabitant and the 4-digit postal code. Sometimes a certain postal code was found in several neighborhoods. In those cases, a weighted average income for that postal code has been calculated. No data were available for neighborhoods with less than 100 inhabitants. Percentiles were calculated on the average income per inhabitant over all neighborhoods in the Netherlands. Then, the patients were classified into income categories: low (lowest 30%), medium (middle 40%), high (highest 30%) or unknown (no data available).

**Table 1:** Overview collected procedures.

**Diagnostics**
• Routine oral health examinations (ROEs)
• Incidental consultations
• Intraoral radiographs (bitewing radiographs)
**Prevention**
• Sessions involving oral hygiene instruction
• Sessions involving professional tooth cleaning
• Sessions involving fluoride treatment
• Sessions involving 1 or more sealants
**Curation**
• Session involving Non-Operative Caries Treatment and Prevention in the primary dentition (NOCTP)
• Restorations (including prefab crowns for the primary dentition)
• Extractions
• Endodontic treatments
**Orthodontics**
• Orthodontic procedures

### Care patterns

To distinguish the preventive care patterns over 4 to 5 years, the number of sessions per year involving oral hygiene instruction, professional tooth cleaning, fluoride treatment and sealing were added. Then, this was dichotomized per year into prevention yes or no. Based on this, patients were classified into one of 3 preventive care patterns: no prevention (P0), occasional prevention (2 or more years without prevention (P1)) or regular prevention (0 years or 1 year without prevention (P2)). This classification was based on the extent of periodic focus on preventive care, regardless of the type of procedure, for two reasons. Firstly, repetition is important in preventive care. Secondly, as there was uncertainty about the most effective preventive care, dental practices could vary in the kind of preventive care they provided despite a similar focus on prevention [2].

To distinguish the curative care patterns over 4 to 5 years, the number of sessions involving NOCTP, the number of prefab crowns in primary teeth, restorations in primary and permanent teeth and extractions of primary and permanent teeth were added per year. This was also dichotomized per year, into curative treatment(s) yes or no. The number of years with curation were summed and patients were assigned to one of 3 curative care patterns: no curative treatments (C0), curative treatment(s) in 1 year (C1) or curative treatments in several years (C2). This classification was modified from the caries risk categories based on caries-related treatments over a 2-year period: no procedures (low), 1 procedure (elevated), and 2 or more procedures (high) [11].

Subsequently, the 3 preventive and 3 curative care patterns were combined and reduced to 6 combined care patterns. These could show relationships between curative and preventive care patterns in patients and in groups of patients with different demographic characteristics. A certain relationship could be expected between the curative and preventive care provided to a patient. Within the group receiving no curative treatment, a distinction was therefore made between patients who did not versus did receive prevention. Within the groups curative treatment(s) in 1 year or several years, a distinction was made between patients who regularly received preventive care versus patients who did not receive or occasionally received preventive care. This is shown schematically in Table 2.

**Table 2:** Combined longitudinal preventive and curative care patterns.

Curative care pattern	Preventive care pattern		
	no prevention (P0)	occasional prevention (P1) ^a)^	regular prevention (P2) ^b)^
no curative treatments (C0)	C0 / P0	C1 / P1 + P2	
curative treatment(s) in 1 year (C1)	C1 / P0 + P1		C1 / P2
curative treatments in several years (C2)	C2 / P0 + P1		C2 / P2

^a)^ 2 or more years without preventive care

^b)^ 0 years or 1 year without preventive care

### Caries risk

Based on the clinical model of Mettes et al. (2010), a patient’s risk category was assessed by the total number of caries-related procedures in a 2-year period. These included restorations in primary and permanent teeth, prefab crowns in primary teeth, sessions involving NOCTP, and extractions of primary and permanent teeth. Then, the risk category per patient was defined as: no caries-related procedures (low); 1 caries-related procedure (elevated) and 2 or more caries-related procedures (high).

The longitudinal caries risk profile was based on the data from a patient’s first 2 available years in the dataset (beginning) and then based on the last 2 available years (end). Combining the risk category at the beginning with the risk category at the end resulted in 9 longitudinal caries risk profiles (see the table in the results section). Subsequently, these were reduced to 5-fold profiles. These 5-fold profiles indicated the direction in which a patient’s caries risk had moved and hence, indicated stable, improved, or deteriorated oral health. The classification for these profiles was: low at beginning and end, improved to low, elevated at the end, deteriorated to high, and high at beginning and end. The risk profile elevated at the end was an intermediate group where the direction of the caries risk was unclear: from low to elevated, elevated remained elevated, high to elevated. In any case, there was something going on with these patients, they received 1 caries-related procedure in the 2 years at the end. Therefore, they were (still) at elevated risk.

### Study outcomes

Care patterns and caries risk profiles were distinguished among young patients based on dental procedures over a period of at least 4 to a maximum of 5 years. This led to 4 primary outcomes: the distribution across the study population of 1) longitudinal preventive care patterns, 2) longitudinal curative care patterns, 3) combined longitudinal curative and preventive care patterns, and 4) longitudinal caries risk profiles.

There were 5 secondary outcomes: 1) per longitudinal care pattern, demographic characteristics of patients as shown by the mean age, the distribution of boys and girls and the distribution of different average-income neighborhoods, 2) the distribution of longitudinal curative and preventive care patterns per dental practice, 3) per curative care pattern, the mean number of preventive procedures per patient per dental practice, 4) the distribution of longitudinal curative care patterns per longitudinal caries risk profile, 5) per caries risk profile, the percentage of patients receiving a procedure and the mean number of procedures per patient per year.

### Data analyses

Differences in mean age between included and non-included patients were tested with an independent samples t-test, and differences in the distribution of boys and girls, and in the distribution of income categories with Chi-square tests. Differences in demographic characteristics between preventive, curative and combined curative and preventive care patterns were analyzed consecutively. To reveal differences in mean age, one-way ANOVA tests with a post hoc Tukey test were used. To reveal differences in the distribution of boys and girls, as well as in the distribution of income categories, Chi-square tests with post hoc pair-wise z-tests and Bonferroni correction were used. Differences between the caries risk profiles in the percentages of patients receiving a certain procedure were also analyzed with Chi-square tests and Bonferroni-corrected post hoc tests. Differences between the caries risk profiles in the mean number of procedures per patient per year were tested with one-way ANOVA and post hoc Tukey test. Differences with a p-value of less than 0.05 (Bonferroni corrected where applicable) were considered statistically significant. Post hoc tests were only conducted if the overall ANOVA or Chi-square test was statistically significant. SPSS (version 27) was used for all analyses.

## Results

### Patients

A total of 37 GDPs delivered data on the care they provided to 26,216 patients born between 1 January 2000 and 31 December 2012. Of these, 9,987 non-regular patients were excluded, leaving 16,299 patients for inclusion in the analyses. Of them, 79.2% had a follow-up of 5 years and 20.8% had a follow-up of 4 years. Respectively 11.8% and 22.6% of the patients with 5 and 4 years of follow-up had not had a ROE in an intermediate year (see Table 3). The demographic characteristics of the included patients are described in Tables 4 and 5.

**Table 3:** Selection of regular patients based on the pattern of routine oral health examinations (ROEs).

***Patients in selection (N = 16*.*229)***								
**1 or more ROEs in**					**N (%)**		**number of years included in study**	**if no ROE, exclusion of care provided in**
**2013**	**2014**	**2015**	**2016**	**2017**				
✓	✓	✓	✓	✓	11,506	(70.9)	5	n/a
✓		✓	✓	✓	417	(2.6)	5	n/a
✓	✓		✓	✓	478	(2.9)	5	n/a
✓	✓	✓		✓	459	(2.8)	5	n/a
✓	✓	✓	✓		801	(4.9)	4	2017
✓		✓	✓		137	(0.8)	4	2017
✓	✓		✓		144	(0.9)	4	2017
	✓	✓	✓	✓	1,947	(12.0)	4	2013
	✓		✓	✓	189	(1.2)	4	2013
	✓	✓		✓	151	(0.9)	4	2013
***Patients not in selection (N = 9*.*987)***								
**1 or more ROEs in**								
3 consecutive years or 3 years with a gap of 2 consecutive years					2,590		0	n/a
2 years					2,758		0	n/a
1 year					3,218		0	n/a
not any year					1,421		0	n/a

**Table 4:** Distribution of longitudinal care patterns and mean age per care pattern.

*care pattern*	*N*		*% of total*	*mean age Jan*. *2013 (sd)*	
total	16,229		(100)	6.8 (3.5)	
**Distribution of demographic characteristics per preventive care pattern**					
P0—not any	1,492	(9.2)		4.7 (4.0)	^b,c^
P1—occasionally	5,704	(35.1)		5.3 (3.5)	^a,c^
P2—regularly	9,033	(55.7)		8.0 (2.7)	^a,b^
**Distribution of demographic characteristics per curative care pattern**					
C0—not any	6,758	(41.6)		6.2 (3.7)	^e,f^
C1—in 1 year	4,296	(26.5)		7.3 (3.3)	^d^
C2—in >1 year	5,175	(31.9)		7.2 (3.1)	^d^
**Distribution of demographic characteristics per combination of curative and preventive care pattern**					
C0 / P0	936	(5.8)		3.9 (3.8)	^h,i,j,k,l^
C0 / P1+P2	5,822	(35.9)		6.5 (3.5)	^g,i,j,k,l^
C1 / P0+P1	1,773	(10.9)		5.8 (3.7)	^g,h,j,l^
C1 / P2	2,523	(15.5)		8.4 (2.6)	^g,h,i,k,l^
C2 / P0+P1	1,819	(11.2)		5.9 (3.4)	^g,h,j,l^
C2 / P2	3,356	(20.7)		7.8 (2.7)	^g,h,i,j,k^

Statistically significant difference in mean age on the 0.05 level between this care pattern and

^a^ P0, ^b^ P1, ^c^ P2. ^d^ C0, ^e^ C1, ^f^ C2, ^g^ C0 / P0, ^h^ C0 / P1+P2, ^i^ C1 / P0+P1, ^j^ C1 / P2, ^k^ C2 / P0+P1, ^l^ C2 / P2.

**Table 5:** Distribution of gender and average income neighborhood per longitudinal care pattern.

*care pattern*	*N*	*gender*				*average income in neighborhood*												
		*boys*		*girls*		*low*			*medium*			*high*			*unknown*			
		*N*	*%)*	*N*	*(%)*	*N*	*(%)*		*N*	*(%)*		*N*	*(%)*		*N*	*(%)*		
total	16,229	8,200	(50.5)	8,029	(49.5)	5,057	(31.2)		6,857	(42.3)		4,089	(25.2)		226	(1.4)		
**Distribution of demographic characteristics per preventive care pattern**																		
P0—not any	1.492	743	(49.8)	749	(50.2)	432	(29.0)	^b,c^	767	(51.4)	^a,c^	273	(18.3)	^a,b^	20	(1.3)		
P1—occasionally	5.704	2,811	(49.3)	2,893	(50.7)*	1,847	(32.4)	^d^	2,412	(42.3)	^d^	1,388	(24.3)	^d^	57	(1.0)		^a,b,c^
P2—regularly	9.033	4,646	(51.4)	4,387	(48.6)*	2,778	(30.8)	^c,d^	3,678	(40.7)	^c,d^	2,428	(26.9)	^a,b^	149	(1.6)		^a,b^
**Distribution of demographic characteristics per curative care pattern**																		
C0—not any	6.758	3,444	(51.0)	3,314	(49.0)	1,799	(26.6)	^b,c^	3,038	(45.0)	^a,d^	1,845	(27.3)	^a,d^	76	(1.1)		^b,c^
C1—in 1 year	4.296	2,131	(49.6)	2,165	(50.4)	1,359	(31.6)		1,772	(41.2)		1,108	(25.8)		57	(1.3)		
C2—in >1 year	5.175	2,625	(50.7)	2,550	(49.3)	1,899	(36.7)	^b,c^	2,047	(39.6)	^a,d^	1,136	(22.0)	^a,d^	93	(1.8)		^b,c^
**Distribution of demographic characteristics per combination of curative and preventive care pattern**																		
C0 / P0	936	465	(49.7)	471	(50.3)	269	(28.7)	^b^	466	(49.8)	^a,c^	186	(19.9)	^b^	15	(1.6)		
C0 / P1+P2	5.822	2,979	(51.2)	2,843	(48.8)	1,530	(26.3)	^b,c^	2,572	(44.2)	^a,c,d^	1,659	(28.5)	^a,b,d^	61	(1.0)		^b,c^
C1 / P0+P1	1.773	853	(48.1)	920	(51.9)	584	(32.9)	^c^	782	(44.1)	^c^	387	(21.8)	^a,b^	20	(1.1)		
C1 / P2	2.523	1,278	(50.7)	1,245	(49.3)	775	(30.7)	^c^	990	(39.2)	^c^	721	(28.6)	^a,b^	37	(1.5)		
C2 / P0+P1	1.819	909	(50.0)	910	(50.0)	676	(37.2)	^b,c^	764	(42.0)	^a,c^	352	(19.4)	^a,b^	27	(1.5)		
C2 / P2	3.356	1,716	(51.1)	1,640	(48.9)	1,223	(36.4)	^b,c^	1,283	(38.2)	^a,d^	784	(23.4)	^a,d^	66	(2.0)		^b,c^

* Statistically significant difference in the distribution of boys and girls on the 0.05 level in P1 and in P2

Statistically significant difference on the 0.05 level, per care pattern (per row) between this category for average income neighborhood and ^a^ low-income neighborhoods, ^b^ medium-income neighborhoods, ^c^ high-income neighborhoods, ^d^ unknown-income neighborhoods.

A comparison between included and non-included patients showed that the included patients were on average somewhat older on 1 January 2013 (6.8 (SD 3.5) versus 5.3 (SD 3.9)), were less likely to live in a neighborhood with low incomes (31.6% vs. 38.7%), were more likely to live in a neighborhood with middle incomes (42.3% vs. 35.3%), and were less frequently a boy (50.5% vs. 52.0%).

### Care patterns

Table 4 shows the distribution of preventive, curative, and the combined curative and preventive care patterns. Almost one in ten patients (9.2%) did not get any preventive care (P0), 41.6% received no curative treatments (C0) and 5.8% of patients had had neither preventive care nor curative treatments (C0/P0). Most patients (55.7%) received preventive care almost annually (P2). The most common combined care pattern (35.9%) was no curative treatments but prevention (C0/P1+P2), followed by (20.7%) the combination curative treatments in several years and regular prevention (C2/P2).

The mean age differed over the preventive, curative, and combined patterns, respectively (for all p<0.01). Table 4 shows that the mean age per care pattern differed between all 3 preventive care patterns. The older the patients, the more prevention they got. Patients without curative treatment(s) (C0) were younger on average than those who had curative treatments in 1 or several years (C1 and C2). The mean age differed between all combined care patterns except between curative treatment(s) in 1 year combined with no or occasional preventive care (C1/P0+P1) and curative treatments in several years combined with no or occasional preventive care (C2/P0+P1). Patients who received no curative treatments and no prevention (C0/P0) were the youngest on average. In all combined care patterns, patients who regularly received prevention were older than those who received no or occasional prevention.

The distribution of boys and girls differed only between the preventive care patterns (p<0.05). Table 5 shows that girls were relatively overrepresented in the care pattern occasional preventive care and boys in regular preventive care. The distributions of patients from different average-income neighborhoods were different between preventive, between curative and between combined care patterns, respectively (for all p<0.01). Patients from medium-income neighborhoods were relatively overrepresented in the care pattern no prevention (P0) and patients from high-income neighborhoods in regular preventive care (P2). Most patients from medium and high-income neighborhoods had no curative treatments (C0), most patients from low-income neighborhoods had curative treatments in several years (C2). The distribution of the average-income neighborhood categories across curative treatment(s) in 1 year (C1) was as expected. Furthermore, patients from low-income neighborhoods were relatively overrepresented in the combined care pattern curative treatments in several years and regular preventive care (C2/P2), patients from medium-income neighborhoods were relatively overrepresented in no curative treatment(s) and no preventive care (C0/P0), and patients from high-income neighborhoods were relatively overrepresented in curative treatment(s) in 1 year and regular preventive care (C1/P2).

### Provided care per dental practice

**Fig 1:**
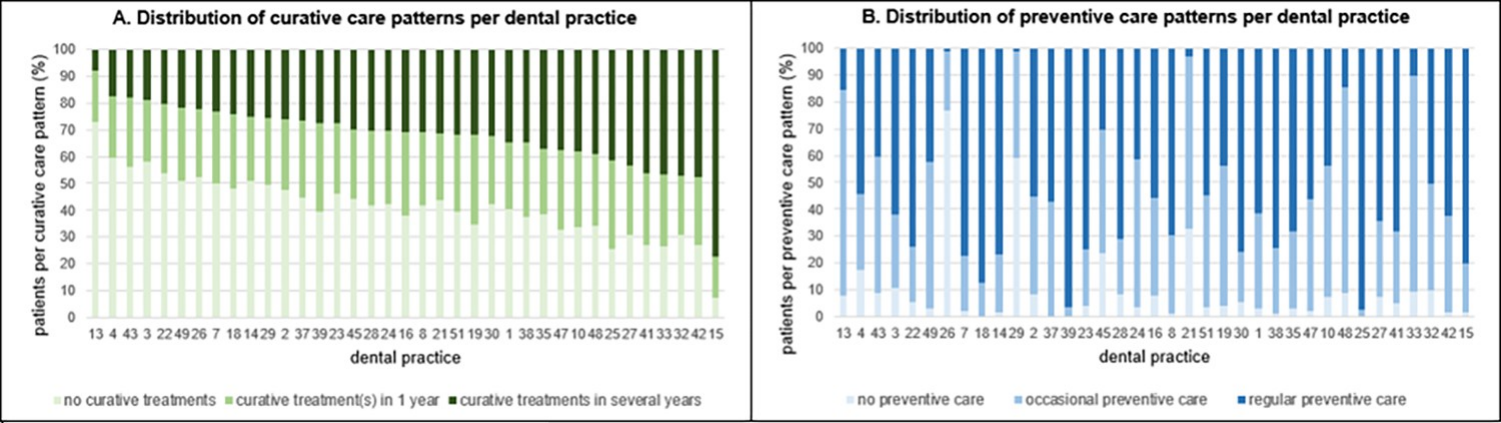
Distribution of curative and preventive care patterns per dental practice (n = 37). The percentages of patients with no curative treatments and patients with curative treatment(s) in 1 year were added and dental practices were then ranked in descending order (Fig 1A). The dental practices in Fig 1B are shown in the same order.

**Fig 2:**
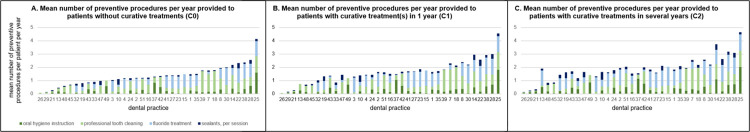
Mean number of preventive procedures per curative care pattern per dental practice (n = 37). The mean number of 4 preventive procedures were added and dental practices were then ranked in ascending order (Fig 2A). The dental practices in Fig 2B and 2C are shown in the same order.

Fig 1 shows the distribution of curative and preventive care patterns per dental practice. There was variation in the distribution of curative care patterns between dental practices and thus in the caries-related care provided to their patient population. The distribution of preventive care patterns per dental practice, arranged in the same order, then showed that there was no relationship between curative and preventive care patterns at the practice level. Despite a patient’s curative care pattern, some dental practices provided no prevention or occasional preventive care (such as dental practices 21, 26 and 29), while other dental practices regularly provided preventive care to all their patients (such as dental practices 25 and 39). Fig 2 shows the provided preventive care per dental practice (ranked in ascending order) to patients in the various curative care patterns.

### Caries risk

Based on the first 2 years in the data set (beginning), the caries risk of 65.5% of the patients analyzed was low, of 13.5% elevated and 21.1% high. In total, the caries risk decreased in 19.7% of the patients, remained the same in 56.3% and increased in 24.1%. So, after 2 or 3 years of follow-up, the caries risk of 60.8%, 15.3% and 24.0% of the patients was respectively low, elevated, and high.

The largest group of patients (45.2%) had a low caries risk at the beginning and at the end of the study period. In 15.6% of the patients the caries risk decreased to low (7.3% was elevated at the beginning and 8.3% was high), and in 4.0% the caries risk decreased from high to elevated.

A small minority of patients (8.7%) had a high caries risk at the beginning and at the end of the study period. In 15.3% of the patients the caries risk increased to high (11.4% was low at the beginning, 3.8% was elevated), and in 8.9% the caries risk increased from low to elevated (see Table 6).

**Table 6:** Distribution of caries risk categories at the beginning and end of the study period and of longitudinal caries-risk-profiles over 4 to 5 years; 9- fold and 5-fold (n = 16,229).

caries-risk category beginning, and end based on first 2 years and last 2 years in dataset						longitudinal caries-risk-profile; 9-fold				longitudinal caries-risk profile; 5-fold		
beginning	n	(%)	end	n	(%)	beginning	end	n	(%)	course	n	(%)
						low	low	7,337	(45.2)	low at beginning and end	7,337	(45.2)
low	10,629	(65.5)	low	9,864	(60.8)	elevated	low	1,179	(7.3)	improved to low	2,527	(15.6)
						high	low	1,348	(8.3)			
						high	elevated	657	(4.0)	elevated at the end	2,476	(15.3)
elevated	2,183	(13.5)	elevated	2,476	(15.3)	elevated	elevated	382	(2.4)			
						low	elevated	1,437	(8.9)			
						low	high	1,855	(11.4)	deteriorated to high	2,477	(15.3)
high	3,417	(21.1)	high	3,889	(24.0)	elevated	high	622	(3.8)			
						high	high	1,412	(8.7)	high at beginning and end	1,412	(8.7)

### Longitudinal curative care patterns and caries risk profiles

**Fig 3:**
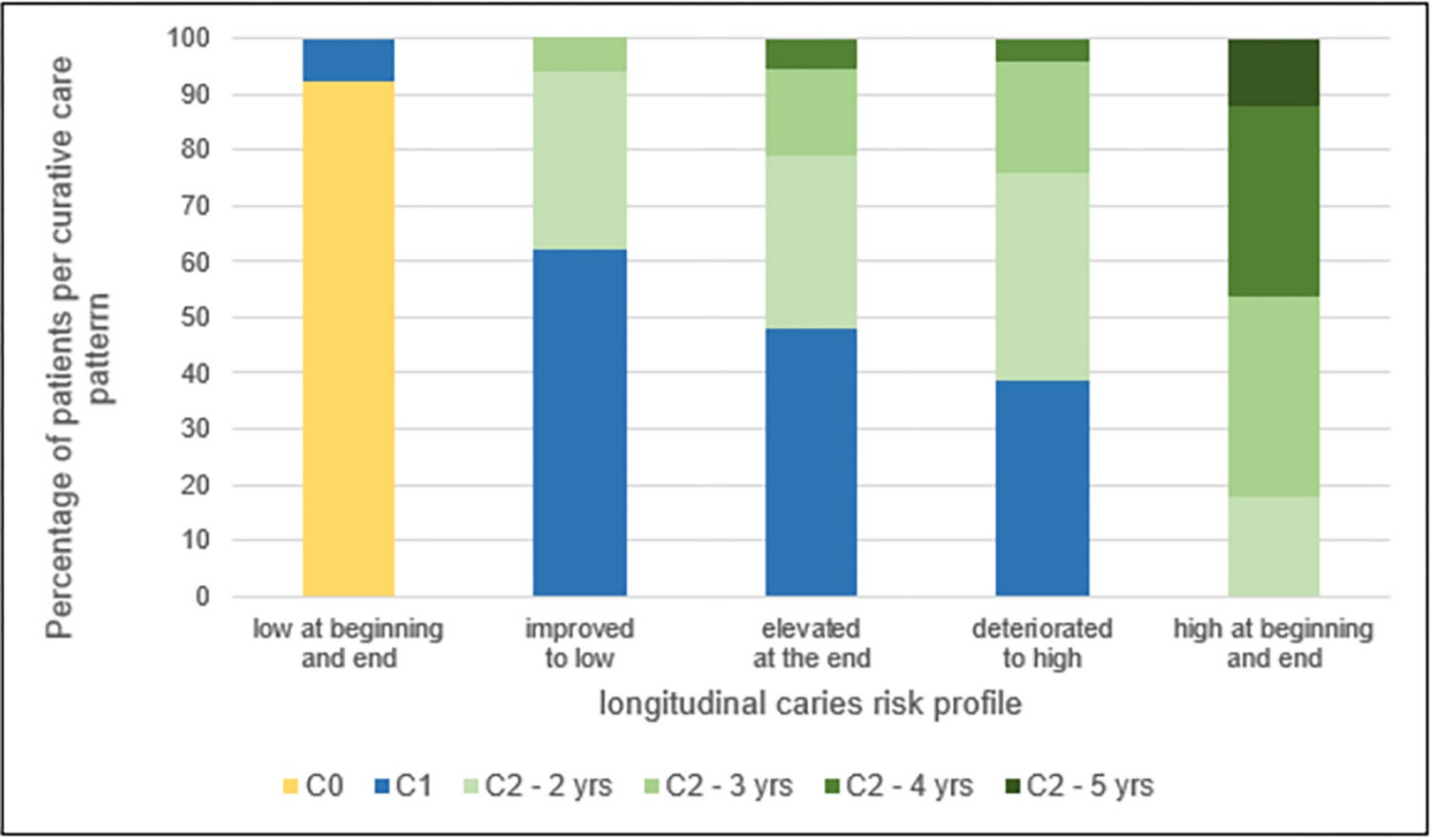
Distribution of longitudinal curative care patterns per longitudinal caries risk profile. C0 is no curative treatments; C1 is curative treatment(s) in 1 year; C2 is curative treatments in several years, subdivided into 2, 3, 4 and 5 years. The difference in the distribution of longitudinal curative care patterns (C0, C1, and subdivided C2) between longitudinal caries risk profiles was statistically significant: Chi-squared tested, p < 0,001.

Fig 3 shows the distribution of longitudinal curative care patterns per longitudinal caries risk profile. Low at beginning and end was the only risk profile involving no curative treatment(s) (C0), in addition, a small group (7.9%) had curative treatment(s) in 1 year (C1). This concerned patients who were included 5 years and received caries-related treatment(s) in the intermediate year (3rd year in dataset).

The risk profiles improved to low, elevated at the end and deteriorated to high showed a mix of curative treatment(s) in 1 year (C1) and several years (C2). Thereby, the distribution shifted more and more towards curative treatments in several years (C2) and within this care pattern the number of years with curative treatment also increased.

The risk profile high at beginning and end consisted exclusively of patients who had had curative treatments in several years (C2) and 12.2% of them received curative treatments in all 5 study years.

### Provided care per longitudinal caries risk profile

Table 7 describes the mean number of diagnostic, preventive, and curative OHS per patient per year for each 5-fold longitudinal caries risk profile. The most frequently conducted preventive procedure was professional tooth cleaning. Oral hygiene instructions, fluoride treatments, and sessions involving sealants were provided least often to patients in caries risk profile low at beginning and end, and most often to patients in risk profile high at beginning and end. Professional tooth cleaning was done mostly in patients whose caries risk improved to low. The spread of the care provided within the various caries risk profiles was large.

**Table 7:** Percentage of patients that received a procedure 1 or more times during the study period and mean number of procedures per patient per year per longitudinal caries-risk-profile.

	Longitudinal caries-risk profile										
procedure	low at beginning and end		improved to low		elevated at the end		deteriorated to high		high at beginning and end		total
**ROE** ^1)^											
- mean number (sd) *	1.57 (0.34)	^b,c,d,e^	1.60 (0.34)	^a^	1.61 (0.34)	^a^	1.59 (0.34)	^a^	1.62 (0.36)	^a^	**1.59 (0.34)**
**incidental consultation**											
- % of patients **	15.0	^b,c,d,e^	26.3	^a,e^	25.2	^a,e^	27.8	^a,e^	35.0	^a,b,c,d^	**22.0**
- mean number (sd) *	0.05 (0.14)	^b,c,d,e^	0.09 (0.19)	^a,e^	0.08 (0.17)	^a,d,e^	0.10 (0.20)	^a,c,e^	0.13 (0.23)	^a,b,c,d^	**0.07 (0.17)**
**intraoral radiograph**											
- % of patients *	32.2	^b,c,d,e^	52.2	^a,c,d^	46.3	^a,b,e^	46.5	^a,b,e^	56.7	^a,c,d^	**41.8**
- mean number (sd) *	0.18 (0.30)	^b,c,d,e^	0.30 (0.35)	^a,c,d,e^	0.27 (0.35)	^a,b,e^	0.27 (0.36)	^a,b,e^	0.39 (0.43)	^a,b,c,d^	**0.24 (0.35)**
**oral hygiene instruction**											
- % of patients **	45.2	^b,c,d,e^	57.1	^a,e^	55.2	^a,e^	56.4	^a,e^	63.3	^a,b,c,d^	**51.9**
- mean number (sd) *	0.22 (0.35)	^b,c,d,e^	0.34 (0.46)	^a,c,e^	0.30 (0.42)	^a,b,e^	0.32 (0.44)	^a,e^	0.43 (0.53)	^a,b,c,d^	**0.28 (0.42)**
**professional tooth cleaning**											
- % of patients **	66.0	^b,c^	76.3	^a,c,d,e^	69.8	^a,b^	67.9	^b^	69.5	^b^	**68.8**
- mean number (sd) *	0.60 (0.64)	^b^	0.73 (0.67)	^a,c,d,e^	0.63 (0.64)	^b^	0.60 (0.64)	^b^	0.63 (0.66)	^b^	**0.63 (0.65)**
**fluoride treatment**											
- % of patients **	50.8	^b,c,d,e^	66.3	^a,e^	63.1	^a,e^	65.4	^a,e^	78.0	^a,b,c,d^	**59.7**
- mean number (sd) *	0.47 (0.61)	^b,c,d,e^	0.71 (0.68)	^a,c,d,e^	0.61 (0.64)	^a,b,e^	0.59 (0.61)	^a,b,e^	0.83 (0.65)	^a,b,c,d^	**0.58 (0.64)**
**sealant(s),** per session											
- % of patients **	35.1	^b,c,d,e^	47.9	^a,e^	48.3	^a,e^	45.4	^a,e^	56.6	^a,b,c,d^	**42.6**
- mean number (sd) *	0.10 (0.18)	^b,c,d,e^	0.16 (0.22)	^a,d,e^	0.15 (0.20)	^a,e^	0.14 (0.20)	^a,b,e^	0.19 (0.23)	^a,b,c,d^	**0.13 (0.20)**
**NOCTP,** per session ^2)^											
- % of patients **	0.1	^b,c,d,e^	8.3	^a,c,d^	3.9	^a,b,d,e^	2.3	^a,b,c,e^	10.2	^a,c,d^	**3.2**
- mean number (sd) *	0.00 (0.01)	^b,c,d,e^	0.02 (0.07)	^a,c.d.e^	0.01 (0.06)	^a,b,d,e^	0.01 (0.04)	^a,b,c,e^	0.03 (0.12)	^a,b,c,d^	**0.01 (0.06)**
**restorations** ^3)^											
- % of patients **	5.4	^b,c,d,e^	73.6	^a,c,d,e^	84.4	^a,b,d,e^	89.3	^a,b,c,e^	98.9	^a,b,c,d^	**49.0**
- mean number (sd) *	0.02 (0.08)	^b,c,d,e^	0.37 (0.42)	^a,c,d,e^	0.41 (0.42)	^a,b,d,e^	0.74 (0.59)	^a,b,c,e^	1.65 (0.99)	^a,b,c,d^	**0.38 (0.64)**
**tooth extraction**											
- % of patients **	3.0	^b,c,d,e^	44.4	^a,c,d,e^	37.4	^a,b,e^	37.6	^a,b,e^	56.7	^a,b,c,d^	**24.7**
mean number (sd) *	0.01 (0.06)	^b,c,d,e^	0.17 (0.27)	^a,c,e^	0.12 (0.20)	^a,b,d,e^	0.17 (0.28)	^a,c,e^	0.30 (0.39)	^a,b,c,d^	**0.10 (0.23)**
**endodontic treatment**											
- % of patients **	0.1	^b,c,d,e^	1.5	^a,e^	2.3	^a,e^	2.4	^a,e^	5.6	^a,b,c,d^	**1.5**
- mean number (sd) *	0.00 (0.01)	^b,c,d,e^	0.00 (0.03)	^a,e^	0.01 (0.03)	^a,e^	0.01 (0.04)	^a,e^	0.01 (0.07)	^a,b,c,d^	**0.00 (0.03)**
**orthodontic procedure** ^4)^											
- % of patients **	7.5	^b,c,d,e^	12.3	^a,c^	9.8	^a,b,d^	12.9	^a,c^	10.0	^a^	**9.6**
N patients	7,337		2,527		2,476		2,477		1,412		**16,229**
(%)	(45.2)		(15.6)		(15.3)		(15.3)		(8.7)		**(100)**

The difference in the percentage of patients that had received the procedure and the mean number of procedures per patient per year between the 5 longitudinal caries-risk-profiles was statistically significant

* one-way ANOVA tested, p < 0.001

** Chi-square tested, p < 0.001

^1)^ Routine oral health examination

^2)^ Non-Operative Caries Treatment and Prevention

^3)^ Restorations in the primary and permanent dentition and prefab crowns in the primary dentition

^4)^ 1 or more orthodontic procedures during the study period. Orthodontic treatment provided by another dental practice or by an orthodontic practice was not visible in the data

Statistically significant difference between caries risk profiles on the 0.05 level, per procedure

^a^ between this caries risk profile and low at beginning and end, ^b^ between this caries risk profile and improved to low, ^c^ between this caries risk profile and elevated at the end, ^d^ between this caries risk profile and deteriorated to high, ^e^ between this caries risk profile and high at beginning and end.

## Discussion

To our knowledge, this is the first observational study in which longitudinal caries-related care patterns provided to Dutch young patients have been described. To do this, longitudinal preventive and curative care patterns were distinguished over a period of 4 to 5 years and analyzed. These patterns were based on the frequency of provided care to either prevent or treat caries over the years, regardless of the specific procedure. The combination of preventive and curative care patterns showed that most young patients did not receive curative treatment(s) (C0) but received occasional or regular preventive care (P1+P2). Patients from high-income neighborhoods got more prevention and patients from low-income neighborhoods more curative treatments. These health differences between children with a high and low socio-economic position are known from the literature [15] and have also been found in periodic epidemiological studies commissioned by the National Health Care Institute [16]. Nevertheless, the differences in provided preventive care are remarkable because the costs are covered for everyone up to the age of 17 from the standard health insurance.

There was considerable variation between dental practices in the distribution of curative and preventive care patterns and there was no relationship between both patterns. Even when looking at the provided preventive care separately for each curative care pattern, there was considerable variation. Dental practices often applied the same preventive approach to most of their patients. This is very similar to supply-sensitive care, which means that the (frequency of the) provided care is mainly determined by the care provider and cannot be explained by the health situation of his patients. This can especially occur if evidence-based clinical practice guidelines are barely available [5]. For oral healthcare there were only a few clinical guidelines available indeed and the fee-per-item system could further encourage unwanted treatment variation [17]. Anyway, uncertainty about the most effective preventive or curative treatment for various types of caries lesions was reason for KIMO to publish the clinical practice guideline Oral healthcare (0–18 yrs) prevention and treatment of dental caries [2]. It is recommended to focus primarily on motivating and training children to promote healthy behavior for good oral health, to administer (additional) fluoride if necessary and to seal therapeutically. If restorative treatment is necessary anyway, it should be minimally invasive.

Obviously, there is a correlation between curative care patterns and caries risk profiles. Both are, in different ways, based on caries-related treatments. Caries risk profiles cover the number of caries-related treatments in blocks of 2 years, one block at the beginning and one at the end of the study period. In those cases that patients were included for 5 years, any caries-related treatments in the intermediate year were therefore not visible. For curative care patterns, only the number of years with curative treatment was considered. This could be 0, 1, or 2 to 5 years. The distribution of the years with curative treatment was not taken into account. For example, 2 years with curative treatments could be completely at the very beginning or end of the period. Nevertheless, there was a clearly distinguishable distribution of curative care patterns per risk profile.

The percentages of patients with improved and deteriorated oral health after 2–3 years of follow-up were in line with the previous findings of Hummel et al. [12]. The goal of oral healthcare, keeping the caries risk low or lowering the caries risk, was achieved in almost two thirds (60.8%) of all patients included. Meanwhile, 15.3% deteriorated to high caries risk and 8.7% remained unchanged at high caries risk. At the same time, 8.9% deteriorated from low to elevated caries risk.

A remarkable finding was that professional tooth cleanings were most often done in patients who improved to low caries risk. It is to be expected that this was only indicated in patients with insufficient self-care, which would then remain a risk factor for the development or progression of caries lesions [18]. Patients who improved to low (15.6%) and patients who had a high caries risk both at the beginning and at the end (8.7%) received the most preventive care. However, the spread was considerable.

In a previous study by Hummel et al. [12], the determination of the risk category was based on claimed restorations. Extractions have now been added to this, resulting in a higher classification of 8% of the patients. The reason for extraction was unknown. This may have led to an overestimation of the number of caries-related treatments because some extractions were probably indicated due to exfoliation problems or had been related to orthodontic treatment. For this reason, extractions of permanent premolars were excluded. Patients who improved to low relatively often had had a tooth extraction. That may have been a non-caries-related tooth extraction at the beginning in an otherwise caries-inactive mouth, so they were misclassified. On the other hand, if extractions were not included, the caries risk would be underestimated. It was assumed that a certain underestimation would give a greater bias than a possible overestimation.

### Limitations

The use of claims data on provided care has some limitations.

- NOCTP was sometimes claimed several times a day for the same tooth. Because the number per claimed procedure was unavailable, it was not known whether this had been carried out or whether it was a correction. That was also a reason to convert the number to 1 per session.
- The indication for treatment was unknown. This could have caused bias. For example, restorations could have been made due to trauma or the filling of an endodontic opening. Extraction of primary teeth could have been indicated due to problems with exfoliation and extraction of premolars due to lack of space. With the corrections made, we have tried to limit these imperfections as much as possible.
- The correction for the number of restorations and extractions on behalf of orthodontic reasons may not have been complete. Partial reattachment of a fixed orthodontic retainer could have been claimed with one-surface-restorations. Those were indistinguishable from caries-related restorations.
- Sometimes, the same treatments were claimed differently. For example, a GDP claimed sealants as restorations, which resulted in an overestimation of the number of restorations claimed by him. Furthermore, the procedure NOCTP was not insured by the standard health insurance. This procedure may have been claimed as oral hygiene instruction and/or fluoride treatment. This would have led to an underestimation of the disease burden and an overestimation of the preventive efforts.
- This study concerned the care provided within the participating dental practices. Therefore, there was no data on the oral care that patients received elsewhere. This applied to emergency care in another dental practice and care for which a patient was referred to another dental practice. The latter may have concerned a referral to a dental hygienist, orthodontist, or a pediatric dentist. Despite this limitation, it can be stated that the delivered data on OHS provided to the patients involved in this study has been sufficiently to be able to distinguish care patterns. Even more so because in the Dutch situation almost all OHS for young patients is generally provided within a single dental practice.

A strength of this study was the inclusion of a large number of patients. However, they were from a limited number of dental practices (N = 37). That, and the unknown clinical situation of the patients did not allow causal analyses and estimates. Therefore, this study only had a descriptive approach.

### Relevance

This study was based on data over the period 2013–2017. Since then, no changes have been made to the claims system nor to the existing reimbursements in the standard health insurance. It can therefore be assumed that the results still reflect the care patterns that are being provided to young patients today. However, in recent years more and more GDPs have been implementing the NOCTP method (like the Nexø method) and after the study period, in 2020, a guideline was published for the prevention and treatment of dental caries in young patients [2]. Their use in practice may lead to a different treatment approach, although the publication of a guideline does not automatically lead to its application [19].

This study was conducted in the Netherlands, but the results offer the opportunity for international reflections as the described methods can also be applied abroad. Either based on claims data in countries with a payment system of fee-per-item or based on patient files in other countries.

The care patterns described in this study, mainly showed the extent to which GDPs provided frequent preventive and curative care. We lumped all preventive procedures together as well as all curative procedures. Our results clearly showed variation in the frequency of the provision of this care between GDPs and this could be a starting point for peer discussions. More insight in the frequency of the underlying procedures described in Table 1 would further deepen such discussions.

The combinations of longitudinal preventive and curative care patterns offer insights into the treatment approaches of GDPs and the extent to which they focus on the caries risk of a patient. The classification of patients into 5 longitudinal caries risk profiles can offer GDPs insight into the development of the oral health of their patient population and outcomes of their care. Furthermore, relating the longitudinal curative care patterns and caries risk profiles to the provided prevention can gain insight into the effect of that care and provide a starting point for efficient use of preventive care. Further clinical research is needed to find explanations for changes in caries risk-categories.

Treatment variation implies that the provided care is not always appropriate. Some patients receive less care than necessary, others more [20]. This can result in poorer oral health or unnecessary high costs. Our data did not allow definitive conclusions on under- or overtreatment. The clinical situation of the included patients was unknown before, during, and at the end of the study period. Nonetheless, the conclusion could be made that the provided care had signs of supply-driven care. It is difficult to identify the causes of treatment variation. It is also difficult to distinguish between warranted and unwarranted treatment variation [6]. In this study, only the provided care was known, but its indication was not. It is well known that major differences exist in the Netherlands in the management of caries in the primary dentition (monitoring, non-restorative, or curative treatment like restoration or tooth extraction) [1, 3]. It is also known that GDPs differ in the stage at which they intervene restoratively in case of caries in the permanent dentition [4]. In addition, GDPs may differ in their focus on prevention and their attitude towards the treatment of very young patients. All of this is expected to have an impact on the care provided. Moreover, differences in provided care may be related to characteristics of GDPs, dental practices, and the organization of the care. Further research is needed to gain more insight into the relationship between these factors and the care patterns described here.

### Conclusions

Using longitudinal data on provided OHS, care patterns of GDPs and caries risk profiles of patients could be distinguished. The most common care patterns were regular preventive care, no curative care, and the combined care pattern no curative treatments and occasional or regular preventive care. The last applied to more than a third of the young patients in the study. Comparison of care patterns showed differences in the mean age of patients and in the distribution of patients from neighborhoods with different average incomes. Moreover, comparison of dental practices showed large differences in the distribution of preventive and curative care patterns. And, also, in the mean number of provided preventive procedures within each curative care pattern.

The most common longitudinal caries risk profile was low caries-risk at the beginning and end of the study period, that applied to almost half of the young patients. Longitudinal caries risk profiles had a relationship with longitudinal curative care patterns. There also was a large spread in the provided preventive procedures per patient within the various longitudinal caries risk profiles.

The differences in provided preventive care were related to treatment variation between GDPs. Dental practices seemed to apply the same preventive approach to most of their patients. This is very similar to supply-sensitive care, which means that the provided care is mainly determined by the care provider and cannot be explained solely by the health situation of his patients.
